# Assisted reproductive technology in Japan: A summary report for 2017 by the Ethics Committee of the Japan Society of Obstetrics and Gynecology

**DOI:** 10.1002/rmb2.12307

**Published:** 2019-11-21

**Authors:** Osamu Ishihara, Seung Chik Jwa, Akira Kuwahara, Yukiko Katagiri, Yoshimitsu Kuwabara, Toshio Hamatani, Miyuki Harada, Tomohiko Ichikawa

**Affiliations:** ^1^ Department of Obstetrics and Gynecology Saitama Medical University Saitama Japan; ^2^ Department of Obstetrics and Gynecology Graduate School of Biomedical Sciences Tokushima University Tokushima Japan; ^3^ Department of Obstetrics and Gynecology Faculty of Medicine Toho University Tokyo Japan; ^4^ Department of Obstetrics and Gynecology Nippon Medical School Tokyo Japan; ^5^ Department of Obstetrics and Gynecology School of Medicine Keio University Tokyo Japan; ^6^ Department of Obstetrics and Gynecology Faculty of Medicine The University of Tokyo Tokyo Japan; ^7^ Department of Urology Graduate School of Medicine Chiba University Chiba Japan

**Keywords:** ART registry, freeze‐all strategy, in vitro fertilization, intracytoplasmic sperm injection, Japan Society of Obstetrics and Gynecology

## Abstract

**Purpose:**

The Japan Society of Obstetrics and Gynecology (JSOG) has collected cycle‐based assisted reproductive technology (ART) data in an online registry since 2007. Herein, we present the characteristics and treatment outcomes of ART cycles registered during 2017.

**Methods:**

We collected cycle‐specific information for all ART cycles implemented at participating facilities and performed descriptive analysis.

**Results:**

In total, 448,210 treatment cycles and 56,617 neonates (1 in 16.7 neonates born in Japan) were reported in 2017, increased from 2016; the number of initiated fresh cycles decreased for the first time ever. The mean patient age was 38.0 years (standard deviation 4.6). A total 110,641 of 245,205 egg retrieval cycles (45.1%) were freeze‐all cycles; fresh embryo transfer (ET) was performed in 55,720 cycles. A total 194,415 frozen‐thawed ET cycles were reported, resulting in 66,881 pregnancies and 47,807 neonates born. Single ET (SET) was performed in 81.8% of fresh transfers and 83.4% of frozen cycles, with singleton pregnancy/live birth rates of 97.5%/97.3% and 96.7%/96.6%, respectively.

**Conclusions:**

Total ART cycles and subsequent live births increased continuously in 2017, whereas the number of initiated fresh cycles decreased. SET was performed in over 80% of cases, and ET shifted from using fresh embryos to frozen ones.

## 1 INTRODUCTION

Since the first baby in Japan conceived as a result of in vitro fertilization (IVF) was born in 1983, the number of assisted reproductive technology (ART) cycles has dramatically increased each year. According to the latest report from the International Committee Monitoring Assisted Reproductive Technologies (ICMART), Japan has been one of the largest users of ART worldwide in terms of annual number of treatment cycles performed.^1^

As it is essential to monitor the trend and situation of ART treatments implemented in the country, the Japan Society of Obstetrics and Gynecology (JSOG) began an ART registry system in 1986 and launched an online registration system in 2007. Since then, cycle‐specific information for all ART treatment cycles performed in ART facilities has been collected. The aim of the present report was to describe the characteristics and treatment outcomes of registered ART cycles during 2017 in comparison with previous year.^2^

## 2 MATERIALS AND METHODS

Since 2007, the JSOG has requested all participating ART clinics and hospitals to register cycle‐specific information for all ART treatment cycles. The information includes patient characteristics, information on ART treatment, and pregnancy and obstetric outcomes. Detailed information collected in the registry has been reported previously.^3^ For ART cycles performed between January 1 and December 31, 2017, the JSOG requested registration of the information via an online registry system by the end of November 2018.

Using the registry data for 2017, we performed a descriptive analysis to investigate the characteristics and treatment outcomes of registered cycles. The number of registered cycles for the initiation of treatment, egg retrievals, fresh embryo transfer (ET) cycles, frozen‐thawed embryo transfer (FET) cycles, freeze‐all embryos/oocytes cycles, pregnancies, and neonates were compared with those in previous years. The characteristics of registered cycles and treatment outcomes were described for fresh ET and FET cycles. Treatment outcomes included pregnancy, miscarriage and live birth rates, multiple pregnancies, pregnancy outcomes for ectopic pregnancy, intrauterine pregnancy coexisting with an ectopic pregnancy, artificial abortion, stillbirth, and fetal reduction. Furthermore, the treatment outcomes of pregnancy, live birth, miscarriage, and multiple pregnancy rates were analyzed according to patient age. We also described treatment outcomes for cycles using frozen‐thawed oocytes based on medical indications.

## 3 RESULTS

In Japan, there were 607 registered ART facilities in 2017, of which 606 participated in the ART registration system. A total 586 facilities actually implemented ART treatment in 2017; 19 registered facilities did not implement ART cycles. Trends in the number of registered cycles, egg retrievals, pregnancies, and neonates born as a result of IVF, intracytoplasmic sperm injection (ICSI), and FET cycles from 1985 to 2017 are shown in Table 1. In 2017, 448,210 cycles were registered, and 56,617 neonates were recorded, accounting for 1 in 16.7 neonates born in Japan (the total number of neonates born in Japan was 946,065 in 2017). The total number of registered cycles and neonates born as a result of ART demonstrated an increasing trend from 1985 to 2017. In 2017, for the first time ever, the number of registered cycles for fresh IVF and ICSI decreased from the previous year; registered IVF and ICSI cycles decreased by 3.2% and 2.2%, respectively, from the previous year. The number of FET cycles increased continuously; the number in 2017 was 198,985 (a 3.7% increase from 2016), resulting in 67,255 pregnancies and 48,060 neonates in 2017. Among registered fresh cycles, 63.3% were ICSI. In terms of FET cycles, 188,388 FETs were performed, resulting in 62,749 pregnancies and 44,678 neonates born in 2016.

**Table 1:** Trends in the number of registered initiated cycles, egg retrievals, pregnancies, and neonates according to IVF, ICSI, and frozen‐thawed ET cycles in Japan, 1985‐2017

Year	Fresh cycles												FET cycles^c^			
	IVF^a^						ICSI^b^									
	No. of registered initiated cycles	No. of egg retrieval	No. of freeze‐all cycles	No. of ET cycles	No. of cycles with pregnancy	No. of neonates	No. of registered initiated cycles	No. of egg retrieval	No. of freeze‐all cycles	No. of ET cycles	No. of cycles with pregnancy	No. of neonates	No. of registered initiated cycles	No. of ET cycles	No. of cycles with pregnancy	No. of neonates
1985	1,195	1,195		862	64	27										
1986	752	752		556	56	16										
1987	1,503	1,503		1,070	135	54										
1988	1,702	1,702		1,665	257	114										
1989	4,218	3,890		2,968	580	446							184	92	7	3
1990	7,405	6,892		5,361	1,178	1,031							160	153	17	17
1991	11,177	10,581		8,473	2,015	1,661							369	352	57	39
1992	17,404	16,381		12,250	2,702	2,525	963	936		524	42	35	553	530	79	66
1993	21,287	20,345		15,565	3,730	3,334	2,608	2,447		1,271	176	149	681	597	86	71
1994	25,157	24,033		18,690	4,069	3,734	5,510	5,339		4,114	759	698	1,303	1,112	179	144
1995	26,648	24,694		18,905	4,246	3,810	9,820	9,054		7,722	1,732	1,579	1,682	1,426	323	298
1996	27,338	26,385		21,492	4,818	4,436	13,438	13,044		11,269	2,799	2,588	2,900	2,676	449	386
1997	32,247	30,733		24,768	5,730	5,060	16,573	16,376		14,275	3,495	3,249	5,208	4,958	1,086	902
1998	34,929	33,670		27,436	6,255	5,851	18,657	18,266		15,505	3,952	3,701	8,132	7,643	1,748	1,567
1999	36,085	34,290		27,455	6,812	5,870	22,984	22,350		18,592	4,702	4,247	9,950	9,093	2,198	1,812
2000	31,334	29,907		24,447	6,328	5,447	26,712	25,794		21,067	5,240	4,582	11,653	10,719	2,660	2,245
2001	32,676	31,051		25,143	6,749	5,829	30,369	29,309		23,058	5,924	4,862	13,034	11,888	3,080	2,467
2002	34,953	33,849		26,854	7,767	6,443	34,824	33,823		25,866	6,775	5,486	15,887	14,759	4,094	3,299
2003	38,575	36,480		28,214	8,336	6,608	38,871	36,663		27,895	7,506	5,994	24,459	19,641	6,205	4,798
2004	41,619	39,656		29,090	8,542	6,709	44,698	43,628		29,946	7,768	5,921	30,287	24,422	7,606	5,538
2005	42,822	40,471		29,337	8,893	6,706	47,579	45,388		30,983	8,019	5,864	35,069	28,743	9,396	6,542
2006	44,778	42,248		29,440	8,509	6,256	52,539	49,854		32,509	7,904	5,401	42,171	35,804	11,798	7,930
2007	53,873	52,165	7,626	28,228	7,416	5,144	61,813	60,294	11,541	34,032	7,784	5,194	45,478	43,589	13,965	9,257
2008	59,148	57,217	10,139	29,124	6,897	4,664	71,350	69,864	15,390	34,425	7,017	4,615	60,115	57,846	18,597	12,425
2009	63,083	60,754	11,800	28,559	6,891	5,046	76,790	75,340	19,046	35,167	7,330	5,180	73,927	71,367	23,216	16,454
2010	67,714	64,966	13,843	27,905	6,556	4,657	90,677	88,822	24,379	37,172	7,699	5,277	83,770	81,300	27,382	19,011
2011	71,422	68,651	16,202	27,284	6,341	4,546	102,473	100,518	30,773	38,098	7,601	5,415	95,764	92,782	31,721	22,465
2012	82,108	79,434	20,627	29,693	6,703	4,740	125,229	122,962	41,943	40,829	7,947	5,498	119,089	116,176	39,106	27,715
2013	89,950	87,104	25,085	30,164	6,817	4,776	134,871	134,871	49,316	41,150	8,027	5,630	141,335	138,249	45,392	32,148
2014	92,269	89,397	27,624	30,414	6,970	5,025	144,247	141,888	55,851	41,437	8,122	5,702	157,229	153,977	51,458	36,595
2015	93,614	91,079	30,498	28,858	6,478	4,629	155,797	153,639	63,660	41,396	8,169	5,761	174,740	171,495	56,888	40,611
2016	94,566	92,185	34,188	26,182	5,903	4,266	161,262	159,214	70,387	38,315	7,324	5,166	191,962	188,338	62,749	44,678
2017	91,516	89,447	36,441	22,423	5,182	3,731	157,709	155,758	74,200	33,297	6,757	4,826	198,985	195,559	67,255	48,060

Abbreviations: ET, embryo transfer; FET, frozen‐thawed embryo transfer; IVF, in vitro fertilization; ICSI, intracytoplasmic sperm injection.

a Including gamete intrafallopian transfer.

b Including split‐ICSI cycles.

c Including cycles using frozen‐thawed oocytes.

**Figure 1:**
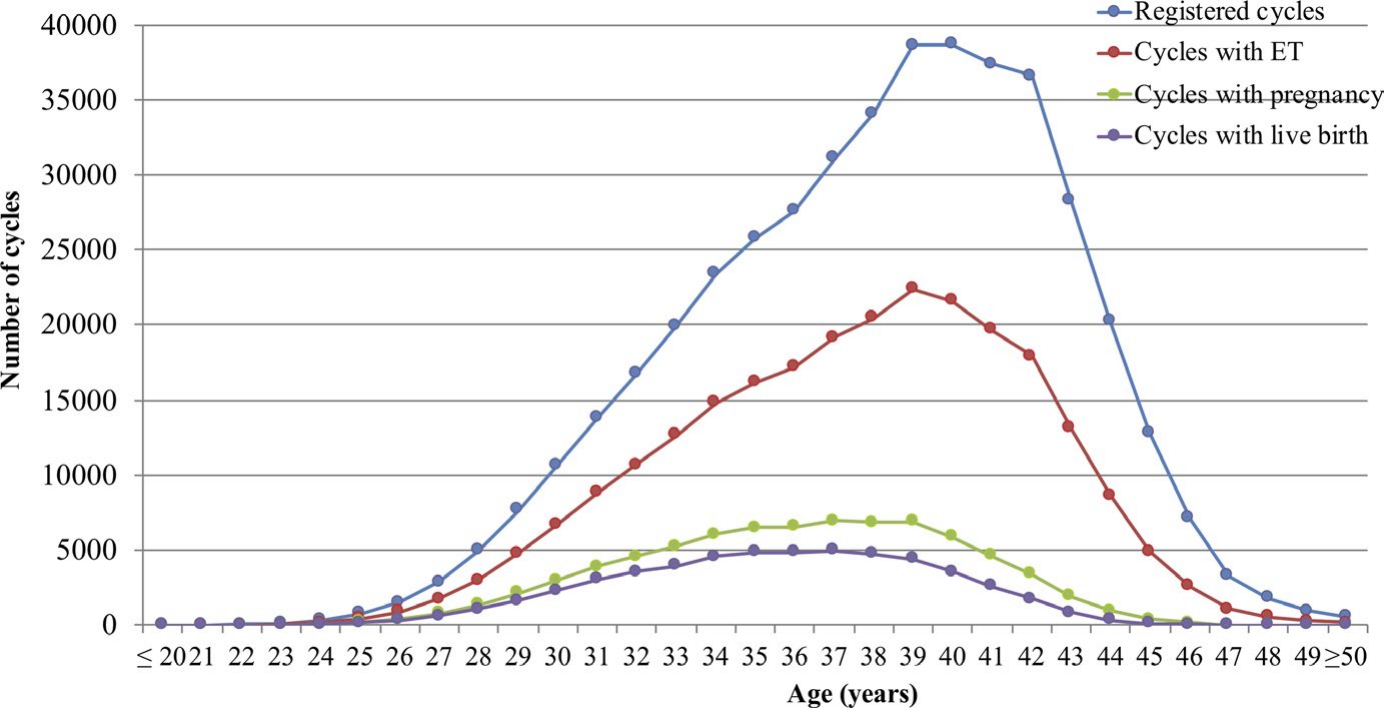
Age distributions of all registered cycles, different subgroups of cycles for ET, pregnancy, and live birth in 2017. Adapted from the Japan Society of Obstetrics and Gynecology ART Databook 2017 (http://plaza.umin.ac.jp/~jsog-art/2017data_20191015.pdf). ET, embryo transfer

The distributions of patient age in registered cycles and different subgroups of cycles with ET, pregnancy, and live birth are shown in Figure 1. The mean patient age for registered cycles was 38.0 years (standard deviation [SD] =4.6); the mean age for pregnancy and live birth cycles was 36.0 years (SD = 4.1) and 35.5 years (SD = 4.0), respectively.

The characteristics and treatment outcomes of registered fresh cycles are shown in Table 2. There were 87,445 registered IVF cycles, 26,485 split‐ICSI cycles, 128,643 ICSI cycles using ejaculated sperm, 2,581 ICSI cycles using testicular sperm extraction (TESE), 25 gamete intrafallopian transfer (GIFT) cycles, 537 cycles with oocyte freezing based on medical indications, and 3,509 other cycles. Of the 245,205 cycles with oocyte retrieval, 110,641 (45.1%) were freeze‐all cycles. The pregnancy rate per ET was 23.0% for IVF and 19.2% for ICSI using ejaculated sperm. Single ET was performed at a rate of 81.8%, with a pregnancy rate of 21.7%. Live birth rates per ET were 16.2% for IVF, 13.2% for ICSI using ejaculated sperm, and 10.2% for ICSI with TESE. The singleton pregnancy rate and live birth rate were 97.5% and 97.3%, respectively.

**Table 2:** Characteristics and treatment outcomes of registered fresh cycles in assisted reproductive technology in Japan, 2017

Variables	IVF‐ET	Split	ICSI		GIFT	Frozen oocyte	Others^a^	Total
			Ejaculated sperm	TESE				
No. of registered initiated cycles	87,445	26,485	128,643	2,581	25	537	3,509	249,225
No. of egg retrieval	85,541	26,185	126,996	2,577	25	530	3,351	245,205
No. of fresh ET cycles	21,939	5,691	26,931	675	25	2	457	55,720
No. of freeze‐all cycles	34,930	17,320	55,585	1,295	0	457	1,054	110,641
No. of cycles with pregnancy	5,047	1,475	5,174	108	7	0	128	11,939
Pregnancy rate per ET	23.0%	25.9%	19.2%	16.0%	28.0%	0	28.0%	21.4%
Pregnancy rate per egg retrieval	5.9%	5.6%	4.1%	4.2%	28.0%	‐	3.8%	4.9%
Pregnancy rate per egg retrieval excluding freeze‐all cycles	10.0%	16.6%	7.2%	8.4%	28.0%	‐	5.6%	8.9%
SET cycles	18,428	4,913	21,502	412	3	‐	300	45,559
Pregnancy following SET cycles	4,234	1,312	4,179	72	0	‐	86	9,883
Rate of SET cycles	84.0%	86.3%	79.8%	61.0%	12.0%	‐	65.6%	81.8%
Pregnancy rate following SET cycles	23.0%	26.7%	19.4%	17.5%	0.0%	‐	28.7%	21.7%
Miscarriages	1,227	327	1,380	36	3	‐	31	3,004
Miscarriage rate per pregnancy	24.3%	22.2%	26.7%	33.3%	42.9%	‐	24.2%	25.2%
Singleton pregnancies^b^	4,755	1,417	4,881	98	7	‐	121	11,279
Multiple pregnancies^b^	114	35	136	4	0	‐	3	292
Twin pregnancies^b^	113	33	134	3	0	‐	3	286
Triplet pregnancies^b^	1	2	2	1	0	‐	0	6
Quadruplet pregnancies^b^	0	0	0	0	0	‐	0	0
Multiple pregnancy rate^b^	2.3%	2.4%	2.7%	3.9%	0.0%	‐	2.4%	2.5%
Live births	3,555	1,085	3,552	69	4	‐	90	8,355
Live birth rate per ET	16.2%	19.1%	13.2%	10.2%	16.0%	‐	19.7%	15.0%
Total number of neonates	3,635	1,103	3,653	70	4	‐	92	8,557
Singleton live births	3,464	1,066	3,439	66	4	‐	88	8,127
Twin live births	84	17	107	2	0	‐	2	212
Triplet live births	1	1	0	0	0	‐	0	2
Quadruplet live births	0	0	0	0	0	‐	0	0
Pregnancy outcomes								
Ectopic pregnancies	58	12	63	0	0	‐	3	136
Intrauterine pregnancies coexisting with ectopic pregnancy	1	0	1	0	0	‐	0	2
Artificial abortions	18	5	25	0	0	‐	0	48
Stillbirths	14	3	14	0	0	‐	1	32
Fetal reductions	1	0	0	1	0	‐	0	2
Unknown cycles for pregnancy outcomes	102	32	111	2	0	‐	2	249

Abbreviations: ET, embryo transfer; IVF‐ET, in vitro fertilization‐embryo transfer; ICSI, intracytoplasmic sperm injection; GIFT, gamete intrafallopian transfer; TESE, testicular sperm extraction; SET, single embryo transfer.

a Others include zygote intrafallopian transfer (ZIFT).

b Singleton, twin, triplet, and quadruplet pregnancies were defined according to the number of gestational sacs in utero.

The characteristics and treatment outcomes of FET cycles are shown in Table 3. There were 197,593 registered cycles, among which FET was performed in 194,415 cycles leading to 66,881 pregnancies (pregnancy rate per FET = 34.4%). The miscarriage rate per pregnancy was 25.9%, resulting in a 23.9% live birth rate per ET. Single ET was performed at a rate of 83.5%, and the singleton pregnancy and live birth rates were 96.7% and 96.6%, respectively.

**Table 3:** Characteristics and treatment outcomes of frozen‐thawed embryo transfer cycles in assisted reproductive technology, Japan, 2017

Variables	FET	Others^a^	Total
No. of registered initiated cycles	197,593	1,199	198,792
No. of FET	194,415	1,053	195,468
No. of cycles with pregnancy	66,881	353	67,234
Pregnancy rate per FET	34.4%	33.5%	34.4%
SET cycles	162,343	746	163,089
Pregnancy following SET cycles	57,167	241	57,408
Rate of SET cycles	83.5%	70.8%	83.4%
Pregnancy rate following SET cycles	35.2%	32.3%	35.2%
Miscarriages	17,343	81	17,424
Miscarriage rate per pregnancy	25.9%	22.9%	25.9%
Singleton pregnancies^b^	63,391	324	63,715
Multiple pregnancies^b^	2,168	22	2,190
Twin pregnancies^b^	2,126	22	2,148
Triplet pregnancies^b^	42	0	42
Quadruplet pregnancies^b^	0	0	0
Multiple pregnancy rate^b^	3.3%	6.4%	3.3%
Live births	46 396	228	46 624
Live birth rate per FET	23.9%	21.7%	23.9%
Total number of neonates	47,807	235	48,042
Singleton live births	44,820	213	45,033
Twin live births	1,471	11	1,482
Triplet live births	15	0	15
Quadruplet live births	0	0	0
Pregnancy outcomes			
Ectopic pregnancies	354	2	356
Intrauterine pregnancies coexisting with ectopic pregnancy	3	0	3
Artificial abortions	286	3	289
Stillbirths	180	1	181
Fetal reduction	15	0	15
Unknown cycles for pregnancy outcomes	1,901	33	1,934

Abbreviations: FET, frozen‐thawed embryo transfer; SET, single embryo transfer.

a Including cycles using frozen‐thawed oocytes.

b Singleton, twin, triplet, and quadruplet pregnancies were defined according to the number of gestational sacs in utero.

**Figure 2:**
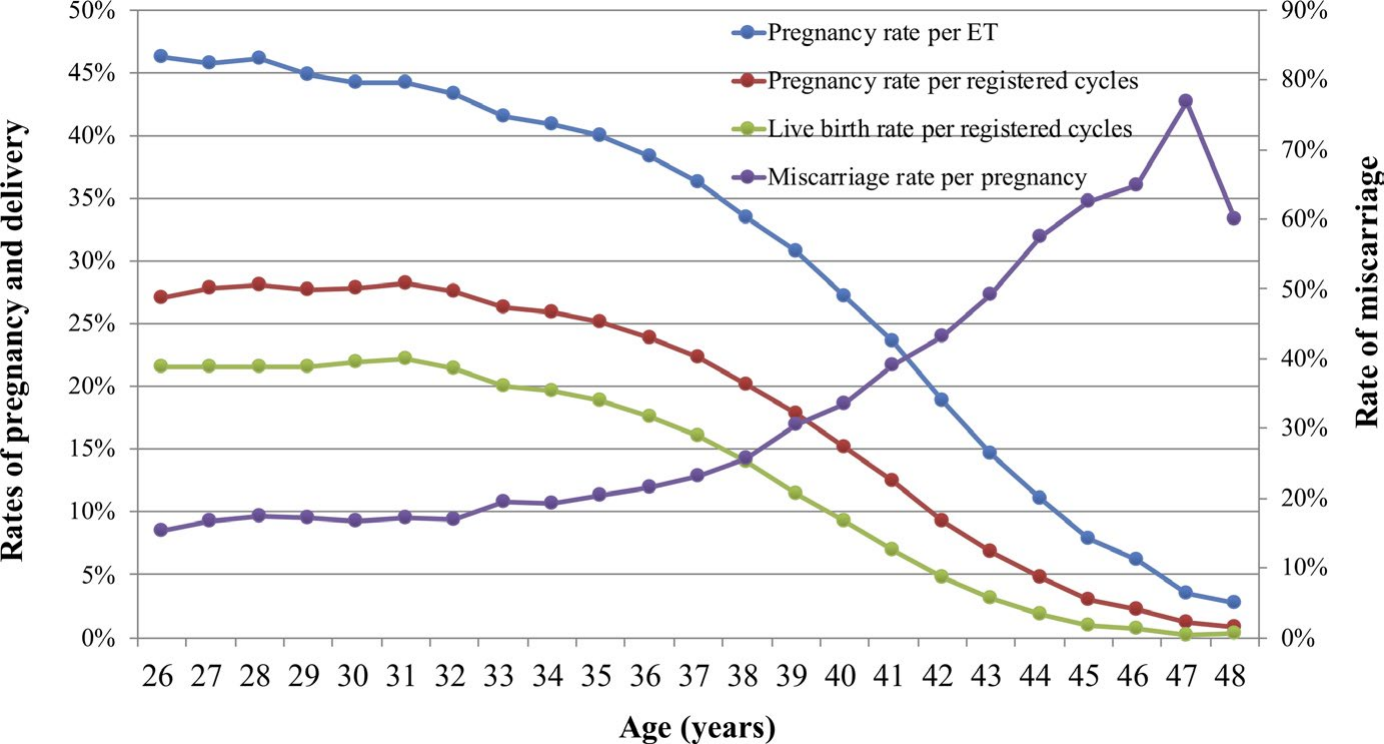
Pregnancy, live birth, and miscarriage rates, according to patient age, among all registered cycles in 2017. Adapted from the Japan Society of Obstetrics and Gynecology ART Databook 2017 (http://plaza.umin.ac.jp/~jsog-art/2017data_20191015.pdf). ET, embryo transfer

Treatment outcomes of registered cycles, including pregnancy, miscarriage, live birth, and multiple pregnancy rates, according to patient age, are shown in Table 4. Similarly, the distribution of pregnancy, live birth, and miscarriage rates according to patient age are shown in Figure 2. The pregnancy rate per ET exceeded 40% up to 35 years of age; this rate gradually fell below 30% after age 39 years and below 10% after age 45 years. The miscarriage rate was below 20% under age 35 years but gradually increased to 33.6% and 49.3% for those aged 40 and 43 years, respectively. The live birth rate per registered cycle was around 20% up to 33 years of age and decreased to 9.3% and 3.1% at ages 40 and 43 years, respectively. Multiple pregnancy rates varied between 2% and 3% across most age groups.

**Table 4:** Treatment outcomes of registered cycles, according to patient age in Japan, 2017

Age (years)	No. of registered initiated cycles	No. of ET cycles	Pregnancy	Multiple pregnancies^a^	Miscarriage	Live birth	Pregnancy rate per ET	Pregnancy rate per registered cycles	Live birth rate per registered cycles	Miscarriage rate per pregnancy	Multiple pregnancy rate^a^
Under 20s	39	10	3	0	1	2	30.0%	7.7%	5.1%	33.3%	0.0%
21	33	12	5	0	1	4	41.7%	15.2%	12.1%	20.0%	0.0%
22	79	37	25	2	5	19	67.6%	31.6%	24.1%	20.0%	8.0%
23	149	86	34	1	7	24	39.5%	22.8%	16.1%	20.6%	2.9%
24	364	199	91	0	10	77	45.7%	25.0%	21.2%	11.0%	0.0%
25	788	468	213	11	46	157	45.5%	27.0%	19.9%	21.6%	5.2%
26	1,569	918	425	15	65	339	46.3%	27.1%	21.6%	15.3%	3.6%
27	2,895	1,762	806	22	134	623	45.7%	27.8%	21.5%	16.6%	2.8%
28	4,965	3,026	1,396	41	242	1,070	46.1%	28.1%	21.6%	17.3%	3.0%
29	7,731	4,792	2,147	55	371	1,671	44.8%	27.8%	21.6%	17.3%	2.6%
30	10,721	6,754	2,990	79	498	2,352	44.3%	27.9%	21.9%	16.7%	2.7%
31	13,829	8,843	3,911	119	673	3,065	44.2%	28.3%	22.2%	17.2%	3.1%
32	16,744	10,678	4,620	143	787	3,596	43.3%	27.6%	21.5%	17.0%	3.2%
33	19,951	12,671	5,259	130	1019	3,988	41.5%	26.4%	20.0%	19.4%	2.5%
34	23,392	14,806	6,055	175	1,172	4,611	40.9%	25.9%	19.7%	19.4%	2.9%
35	25,809	16,210	6,492	247	1,321	4,869	40.0%	25.2%	18.9%	20.3%	3.9%
36	27,594	17,184	6,583	249	1,424	4,867	38.3%	23.9%	17.6%	21.6%	3.9%
37	31,095	19,156	6,940	255	1,612	5,000	36.2%	22.3%	16.1%	23.2%	3.8%
38	34,081	20,484	6,867	224	1,762	4,778	33.5%	20.1%	14.0%	25.7%	3.3%
39	38,618	22,400	6,894	238	2,111	4,440	30.8%	17.9%	11.5%	30.6%	3.5%
40	38,698	21,604	5,872	184	1,973	3,603	27.2%	15.2%	9.3%	33.6%	3.2%
41	37,365	19,672	4,645	146	1,819	2,626	23.6%	12.4%	7.0%	39.2%	3.2%
42	36,600	17,946	3,394	82	1,466	1,763	18.9%	9.3%	4.8%	43.2%	2.5%
43	28,253	13,138	1,932	43	952	881	14.7%	6.8%	3.1%	49.3%	2.3%
44	20,255	8,667	965	17	555	371	11.1%	4.8%	1.8%	57.5%	1.8%
45	12,836	4,956	393	4	246	130	7.9%	3.1%	1.0%	62.6%	1.0%
46	7,147	2,634	165	0	107	54	6.3%	2.3%	0.8%	64.8%	0.0%
47	3,275	1,093	39	0	30	8	3.6%	1.2%	0.2%	76.9%	0.0%
48	1,799	556	15	1	9	5	2.7%	0.8%	0.3%	60.0%	7.1%
49	973	317	14	0	10	3	4.4%	1.4%	0.3%	71.4%	0.0%
Over 50s	563	200	4	0	3	1	2.0%	0.7%	0.2%	75.0%	0.0%

Abbreviation: ET, embryo transfer.

a Multiple pregnancies were defined according to the number of gestational sacs in utero.

The treatment outcomes of cycles using frozen‐thawed oocytes based on medical indications are shown in Table 5. There were a total of 91 cycles using frozen oocytes, among which 21 cycles resulted in pregnancy (pregnancy rate per FET = 23.1%). The miscarriage rate per pregnancy was 14.3%, resulting in a 19.8% live birth rate per ET.

**Table 5:** Treatment outcomes of cycles using frozen‐thawed oocytes based on medical indications in assisted reproductive technology, Japan, 2017

Variables	Embryo transfer using frozen‐thawed oocyte
No. of registered cycles	193
No. of ET	91
No. of cycles with pregnancy	21
Pregnancy rate per ET	23.1%
SET cycles	69
Pregnancy following SET cycles	18
Rate of SET cycles	75.8%
Pregnancy rate following SET cycles	26.1%
Miscarriages	3
Miscarriage rate per pregnancy	14.3%
Singleton pregnancies^a^	20
Multiple pregnancies^a^	1
Twin pregnancies^a^	1
Triplet pregnancies^a^	0
Quadruplet pregnancies^a^	0
Multiple pregnancy rate^a^	4.8%
Live births	18
Live birth rate per ET	19.8%
Total number of neonates	18
Singleton live births	18
Twin live births	0
Triplet live births	0
Quadruplet live births	0
Pregnancy outcomes	
Ectopic pregnancies	0
Intrauterine pregnancies coexisting with ectopic pregnancy	0
Artificial abortions	0
Stillbirths	0
Fetal reduction	0
Unknown cycles for pregnancy outcomes	0

Abbreviations: ET, embryo transfer; SET, single embryo transfer.

a Singleton, twin, triplet, and quadruplet pregnancies were defined according to the number of gestational sacs in utero.

## 4 DISCUSSION

Using the current Japanese ART registry system, we demonstrated that there were a total 448,210 registered ART cycles and 56,617 resultant live births, accounting for 1 in 16.7 neonates born in Japan during 2017, the most since the registry began. However, in 2017, the total number of initiated fresh cycles (both IVF and ICSI) decreased from the previous year for the first time. Freeze‐all cycles predominated, accounting for nearly 45% of total initiated cycles, resulting in frozen cycles accounting for most embryo transfers. The single ET rate was 81.8% for fresh transfers and 83.4% for frozen cycles, which also showed an increasing trend since 2007, reaching a singleton live birth rate of nearly 97% in total. These results represent the latest clinical practice of ART in Japan.

The characteristic that more than 45% of fresh cycles were freeze‐all is unique in Japan. Freeze‐all is beneficial for avoiding complications related to ovarian stimulation, such as ovarian hyperstimulation syndrome (OHSS), especially in high‐risk patients such as those with polycystic ovary syndrome (PCOS) or high ovarian reserve.^4^ However, it remains under discussion whether the freeze‐all strategy is beneficial for the entire IVF population. A recently published meta‐analysis including 11 randomized controlled trials (RCTs) with 5,379 patients reported that freeze‐all and subsequent elective FET demonstrated significantly higher live birth rates than those with fresh ET (risk ratio [RR] =1.12, 95% confidence interval [CI], 1.01 to 1.24) in the entire population.^5^ Interestingly, neither the live birth rate (RR = 1.03, 95% CI, 0.91 to 1.17) in the subgroup of normo‐responders nor the cumulative live birth rate in the entire population (RR = 1.04, 95% CI, 0.97 to 1.11) was significantly different between groups. However, a more recent multicenter RCT investigating the effect of blastocyst ET after freeze‐all or fresh ET cycles among 825 ovulatory women from China demonstrated that a freeze‐all strategy with subsequent elective FET achieved a significantly higher live birth rate than did fresh blastocyst ET (RR = 1.26, 95% CI, 1.14 to 1.41).^6^ Thus, evidence for the use of a freeze‐all strategy in the entire IVF population remains limited.

FET might increase specific complications during pregnancy. Previous analysis using the Japanese ART registry has demonstrated that FET is associated with significantly higher risk for hypertensive disorders of pregnancy (HDP) and placenta accreta than fresh ET.^7^ In particular, higher risk of HDP in FET is noted in several RCTs.^4^, ^6^ Recently, it was reported that methods of endometrium preparation, especially hormone replacement cycles for FET, might be associated with these complications.^8^ Therefore, caution should be exerted with respect to the risks of specific pregnancy complications, although frozen ET is reported to be beneficial in avoiding OHSS and reducing the risk of low birth weight and preterm delivery, as compared with fresh cycles.^9^

ICSI cycles accounted for 63% of registered fresh cycles in 2017. An increasing trend in ICSI use is seen worldwide. The latest report from ICMART indicates that ICSI was used at a rate of 66.5% in 2011 among 65 countries and 2,560 ART clinics.^1^ That report also highlighted that large disparities exist for ICSI; the ICSI rate varies from 97% in Middle Eastern countries to 55% in Asia, 69% in Europe, and 73% in North America. Importantly, despite its increased use, there is no rigorous evidence that ICSI improves reproductive outcomes, especially for non‐male factor infertility such as unexplained infertility,^10^ low ovarian reserve, 11 or advanced maternal age.^12^ Based on this insufficient evidence, practice committees of the American Society for Reproductive Medicine and the Society for Assisted Reproductive Technology have concluded that routine use of ICSI in patients with non‐male factor infertility is not recommended.^13^

The strength of the Japanese ART registry is its mandatory reporting system with a high compliance rate, in cooperation with the government subsidy system. Using this system, nearly all participating ART facilities (606 of 607 facilities) have registered cycle‐specific information.

Nevertheless, several limitations exist in the registry. First, the registry includes only cycle‐specific information; it is very difficult to identify cycles in the same patient using the registry. Under current Japanese ART practice, in which nearly half of initiated cycles are freeze‐all, current indicators such as pregnancy and live birth rate per aspiration cycle would be markedly underestimated. In fact, the abovementioned report from ICMART described that the delivery rate per oocyte aspiration in Japan was the lowest among 65 countries, which could mislead public opinion regarding the quality of treatment as ET was not performed in most included fresh cycles.^1^ It has recently been suggested that the cumulative live birth rate per oocyte aspiration is more suitable when reporting the success rate of IVF outcomes.^14^, ^15^ Yet, the appropriate definitions to be used in calculating the cumulative live birth rate are under discussion. The format of the Japanese ART registry may need to be changed, to report indicators for comparability.

Second, the Japanese ART registry includes unfertilized oocyte freezing cycles only for medically indicated cases, such as fertility preservation in cancer patients; the registry does not include cycles with non‐medical indications. Because no other regulatory measure in reproductive medicine that includes oocyte freezing is enforced in Japan, there is no information available regarding the practices outside this registry.

In conclusion, our analysis of the ART registry during 2017 demonstrated that the total number of ART cycles increased whereas the number of initiated fresh cycles decreased for the first time ever. SET was performed at a rate of more than 80%, resulting in a 97% singleton live birth rate. Although an increasing trend for frozen ET and freeze‐all cycles is characteristic in Japan, further investigation is required to evaluate the effect of the freeze‐all strategy and frozen ET on cumulative live births, and particularly with respect to both maternal and neonatal safety issues. These data represent the latest clinical practices of ART in Japan. Further improvement in the ART registration system in Japan is important.

## CONFLICT OF INTEREST

There is no conflict of interest regarding the publication of this study.

## HUMAN RIGHTS STATEMENT AND INFORMED CONSENT

All procedures were performed in accordance with the ethical standards of the relevant committees on human experimentation (institutional and national) and the Helsinki Declaration of 1964 and its later amendments. Informed consent was obtained from all patients included the study.

## ANIMAL RIGHTS

This report does not contain any studies performed by any of the authors that included animal participants.

## APPROVAL BY ETHICS COMMITTEE

Not applicable.
